# Improving education in primary care: development of an online curriculum using the blended learning model

**DOI:** 10.1186/1472-6920-9-33

**Published:** 2009-06-10

**Authors:** Linda Orkin Lewin, Mamta Singh, Betzi L Bateman, Pamela Bligh Glover

**Affiliations:** 1 Department of Pediatrics, Case Western Reserve University School of Medicine, Cleveland, Ohio, USA; 2 Department of Pediatrics, University of Maryland School of Medicine, Baltimore, Maryland, USA; 3 Department of Internal Medicine, MetroHealth Medical Center/Case Western Reserve University School of Medicine, Cleveland, Ohio, USA; 4 Department of Internal Medicine, Louis Stokes Cleveland Veterans Administration Hospital/Case Western Reserve University School of Medicine, Cleveland, Ohio, USA; 5 Primary Care Track Program, Case Western Reserve University School of Medicine, Cleveland, Ohio, USA; 6 Department of Educational Foundations and Special Services, School of Lifespan Development and Educational Services, Kent State University, Kent, Ohio, USA; 7 Primary Care Track Program, Case Western Reserve University School of Medicine, Cleveland, Ohio, USA; 8 Current address: Macromolecular Science & Engineering, Case Western Reserve University School of Engineering, Cleveland, Ohio, USA

## Abstract

**Background:**

Standardizing the experiences of medical students in a community preceptorship where clinical sites vary by geography and discipline can be challenging. Computer-assisted learning is prevalent in medical education and can help standardize experiences, but often is not used to its fullest advantage. A blended learning curriculum combining web-based modules with face-to-face learning can ensure students obtain core curricular principles.

**Methods:**

This course was developed and used at The Case Western Reserve University School of Medicine and its associated preceptorship sites in the greater Cleveland area. Leaders of a two-year elective continuity experience at the Case Western Reserve School of Medicine used adult learning principles to develop four interactive online modules presenting basics of office practice, difficult patient interviews, common primary care diagnoses, and disease prevention. They can be viewed at . Students completed surveys rating the content and technical performance of each module and completed a Generalist OSCE exam at the end of the course.

**Results:**

Participating students rated all aspects of the course highly; particularly those related to charting and direct patient care. Additionally, they scored very well on the Generalist OSCE exam.

**Conclusion:**

Students found the web-based modules to be valuable and to enhance their clinical learning. The blended learning model is a useful tool in designing web-based curriculum for enhancing the clinical curriculum of medical students.

## Background

Computer assisted instruction in undergraduate medical education has gained popularity in the last two decades. Web-based learning is attractive for many reasons, including the efficiency of providing content at diverse locations, the flexibility that students have in accessing and reviewing content, and the ability to provide online links to related information [1-3]. Computer-aided instruction is particularly attractive to directors of clinical experiences in complying with LCME standards and regulating the curricula of geographically dispersed students who are exposed to a variety of preceptor styles and patient populations [4].

Reports of widespread use of web-based curricula in the medical education literature speak to the convenience and feasibility of this method [5-12]. However, there is little description of the way in which didactic content has been reformatted to take advantage of the electronic learning environment [13-17]. Further, the use of computer-based materials to support clinical learning, a form of "blended learning," is new to medical education and has not been well studied [18-20].

The one study that did address the affect of internet-based learning did not specifically target blended learning, but did focus on the affect internet-based instruction had on the learning of health professional students when compared with no intervention and no internet based educational interventions. The authors performed a meta-analysis and concluded that internet-based instruction, when compared with no intervention, was associated with positive learning outcomes, particularly in learner satisfaction and knowledge acquisition, but in the knowledge arena blended learning courses were excluded from the analysis [21].

At the Case Western Reserve University School of Medicine (Case), the Community Primary Care Preceptorship (CPCP) was created as an elective blended learning program consisting of clinical and online components. Interested students completed a two-year primary care preceptorship in the practice of a general internist, family physician, general pediatrician, or medicine-pediatrics physician in the greater Cleveland area. The online instructional component for this course was created with three goals:

1. To standardize the core curricular concepts for all students.

2. To design online modules based on adult learning principles and present them in a developmentally appropriate manner, with increasing levels of complexity.

3. To take advantage of the flexible and interactive nature of the online environment to engage learners and augment and reinforce learning in the clinical setting.

The purpose of this paper is to describe the online curriculum as originally developed, as well as present student feedback and performance data.

## Methods

### Program Description

The CPCP online curriculum can be viewed at . Learning modules can be found under "For Students." The description of the modules below refers to the content of the CPCP course when it was part of the Case Medical

School's Robert Wood Johnson Foundation funded elective Primary Care Track for students interested in exploring careers in primary care (1994–2007). Since then the CPCP curriculum has been amended to be used by the entire student body at Case, and has also been changed to allow educators at other institutions to use its learning activities. For this reason, the interactive parts of the website, described below, are no longer functional, as they required the ability to login to the Case computer system for students to post real-time comments and for course leaders to respond.

A panel of community primary care physicians who served as medical student preceptors, identified the topics included in the curriculum thus ensuring the online component would reflect real clinical scenarios. The modules revolve around a panel of "patients" that remains constant, each with a life story that addresses a portion of the curriculum. They include a two-year-old child, an adolescent girl, a young adult woman, a middle-aged adult man, and an elderly woman. Each "patient" has an online chart that is updated after each module and a variety of interactive methodologies make the patients and their clinical issues come to life.

Several adult-learning principles guided module development. First, adult learners are self-directed, so modules were constructed so that despite the fixed sequence of the modules themselves, students could complete required components of each module in any order. Also, each module includes links to more detailed information so that students can pursue deeper learning in areas of interest.

Further, adult learners are goal and relevancy-oriented, so the information in each module is presented in its most applicable form. Patient scenarios were created to represent common situations, and the practical aspects of responding to those situations are highlighted. Adult learners also bring a wealth of personal experiences to their learning, so the original CPCP modules used open-ended questions and discussion boards to allow students to address clinical issues from their own perspectives. The answers posted to the discussion board were reviewed by the course director and web instructional designer after the students completed each module and relevant feedback and responses were posted for the group to see. As noted above, in order to make this resource available to a larger academic community, the interactive nature of the discussion board has been removed from the current website.

Module One is called "You the Primary Care Physician," and is completed in the first half of the second year of medical school, when students have had limited exposure to clinical practice. The opening page shows a "virtual office" with staff members in place and a chart visible on a counter. Students can click on office staff to learn about their roles and responsibilities. They can go to the virtual chart and read a complete new patient note, see a properly completed problem list, and access tutorials on writing Subjective, Objective, Assessment and Plan (SOAP) notes and prescriptions. There is also a short health insurance tutorial followed by a quiz. Students were required to submit a SOAP note from their preceptorship sites for feedback from a course leader and two prescriptions written in the correct format. All of the activities are practical and designed to give students additional background and experience in the nuts and bolts of working in a medical setting.

Module Two, "The Art of Medicine," presents topics relating to challenging communication issues including maintaining adolescent confidentiality, addressing hidden agendas, conducting mental status exams in the elderly, confronting patients that miss appointments, and obtaining informed consent. Students enter a virtual waiting room and navigate by clicking on each patient. That patient's scenario is then presented along with audio, video, and text examples of possible responses. When the discussion board was in use, students answered open-ended questions after each scenario reflecting their real experiences in their clinical sites, and responses were posted on a discussion board where other students and faculty members could see them and respond.

Module Three is completed in the first half of the third year, when students are comfortable with the basics and can focus on clinical problem solving. It includes the same five patients presenting with common primary care complaints. Each activity includes a discussion of the differential diagnosis, work-up, and treatment of the highlighted condition as well as a related primary care topic such as over-prescription of antibiotics, complementary and alternative medicine, drug interactions, clinical practice guidelines, and use of online resources in real-time patient care. The content, again, is interactive, allowing students to become part of each scenario.

The final module, "Disease Prevention," takes students out of the medical office and into the patients' homes. It focuses on accidental injuries in children, metabolic syndrome in the obese, diet and exercise in adults, smoking prevention in teens, the secondary complications of diabetes in older adults, and falls in the elderly. It also employs an interactive format to engage students and originally culminated in each student creating a patient education flyer that was saved and could be accessed by any student when needed during real patient care experiences. A summary of each module is shown in Table 1.

**Table 1:** An Overview of the Community Primary Care Preceptorship Modules with Content and Timeline

	**1st Year of CPCP** **2nd Year of CPCP** **Second year of medical school**		**2nd Year of CPCP** **Second year of medical school** **Third year of medical school**	
	**Module 1:** You, the Medicine primary MD	**Module 2:** The Art of	**Module 3:** The Practice of Medicine	**Module 4:** Disease Prevention

**Focus**	Primary care basics	Communicating with the patient	Evidence based medicine in the office setting	Preventable diseases: incidence/prevalence in diverse populations
**Core Content**	Introduction to office-based practice – people and procedures	Difficult patient-communication issues and seeing the patient's point of view	Approaching differential diagnoses	Childhood injury, advanced directives, smoking prevention, metabolic syndrome, secondary complications of diabetes, clinical reminder systems
**Skill Development**	Charting, writing prescriptions, understanding health insurance	Understanding informed consent, performing a mini-mental status exam, maintaining patient confidentiality, addressing noncompliance, identifying hidden agendas	Developing a diagnostic and therapeutic plan for common complaints	Counseling patients for behavior change, performing a foot exam on a diabetic patient, working with other health-care professionals in preventing injury, correctly installing a child safety seat, understanding
**Student Tasks**	Submit a SOAP note, practice prescription writing, and complete a practice profile of preceptorship site	Report on how preceptor handles difficult patient-care communication issues	Review patient cases with common illnesses and identify appropriate care based on clinical evidence	Create a patient education handout written at an appropriate reading level

### Program Evaluation

The CPCP online curriculum was evaluated through feedback from students on the content and function of the modules and their applicability to students' clinical work. Feedback surveys addressed four areas: 1) general information on the time required to complete each module, relevance of the learning objectives, and appropriateness of the online format; 2) applicability of the modules' content to the students' clinical work; 3) module technical performance; and 4) the modules' most and least valuable parts. The first three sections contained Likert scale responses; the questions about the most and least valuable parts of each module were answered in free text. Student responses to the first three sections were tabulated and the percentage indicating each possible response calculated. Comments from section four were reviewed for recurrent themes.

### Student Evaluation

At the end of the third year of medical school, all CPCP students and a convenience sample of students in the traditional curriculum who had no exposure to the CPCP online curriculum or preceptorship completed a 15 station Objective Structured Clinical Examination whose content was based on the CPCP curricular objectives. Stations assessed students' history-taking, physical exam, medical knowledge and patient education skills.

Scores on the exam were collected and analyzed using the SPSS statistical package, v. 14.0. Mean scores on the USMLE Part 1 exam of PCT and control students were compared using ANOVA to look for baseline academic differences between the groups. Levene's test for Equality of Variances was performed on OSCE scores to determine whether the variances between the two groups differed significantly. T-tests were then performed to compare the overall performance of CPCP students and controls, as well as the sub-scores on the history taking, physical exam and patient education stations.

This study was granted "exempt" status by the Institutional Review Board of the MetroHealth Medical Center, a Case affiliate.

## Results

### Program Evaluation

CPCP students from the classes of 2005 (16), 2006 (36), and 2007 (37) completed 248 feedback surveys between January, 2002 and July, 2005. All participating students completed all surveys as a requirement of the program. Analysis was performed on 89 surveys for Module 1, 80 for Module 2, 44 for Module 3, and 35 for Module 4. Variation in the numbers is attributable to changes in some students' status as they joined or left the CPCP program.

Survey results showed that each module, in its entirety, took most students approximately 7–9 hours to complete. Greater than 99% of students stated that the learning objectives of each module were clear and 98% agreed that they were met. Students indicated that they would preferentially choose a web-based format to learn the material in each module (>75% for Modules 1–3; 63% for Module 4) with the majority of remaining students preferring small groups.

The overall content of each module was rated as "excellent" or "very good" by the majority of students. The most highly rated components of each module were those that addressed concrete clinical skills such as SOAP note writing, prescription writing, and the health insurance tutorial (Module 1); adolescent confidentiality and hidden agendas (Module 2); treating childhood asthma and the differential diagnosis of URI symptoms (Module 3); and obesity and smoking prevention (Module 4).

Table 2 shows the students' opinions about whether the module content could be directly related to real patient interactions in their clinical sites, a major goal of employing blended learning. The majority of students agreed or strongly agreed; most applicable were the sections in Module 3 on a patient with an upper respiratory infection (96% strongly agreed or agreed), an asthmatic child (91%), and a patient with diabetes and hypertension (93%).

**Table 2:** Students that Agree or Strongly Agree "I Could Directly Relate what I Learned in the Following Module Components to Real Patient Interactions in my Preceptor's Office"

Module	Component	% Strongly Agree or Agree
Two: The Art of Medicine (n = 50)	Patients who miss appointments*	91
	Obtaining Informed Consent	88
	Finding the Hidden Agenda	74
	The Confidential Adolescent Interview	70
	The Mental Status Exam in the Elderly	60
Three: The Practice of Medicine (n = 44)	URI/Antibiotic Over prescribing	96
	Treatment of Hypertension in a Diabetic Patient	93
	Use of Asthma Guidelines	91
	Working up Knee Pain/CAM	89
	Weight Loss in the Elderly/Using Online Resources	75
Four: Preventive Medicine (n = 14)	Metabolic Syndrome	93
	Smoking Prevention	86
	Secondary Complications of Diabetes	85
	Falls in the Elderly	71
	Patient Education Exercise	71
	Childhood Accidents 50	50

* n = 15 for this component due to error in survey form

The technical performance of all components of all four modules was rated as excellent or very good by >70% of students except for the streaming video in Module 3 (54%) and Module 4 (46%) and the streaming audio in Module 4 (45%). More than 75% of students found the modules easy to navigate and more than 80% found the amount of explanation adequate to perform the required tasks.

### Student Evaluation

A total of 41 CPCP students took the Generalist OSCE exam at the end of their second year of the curriculum corresponding to the end of the third year of medical school. Nine volunteer students comprised the control group.

There were no statistical differences between CPCP students (mean = 224, standard dev = 20.4) and controls (mean = 236, standard dev = 15.1) on the USMLE Part 1 exam.

Levene's test for Equality of Variances indicated that variances between the two groups on the gOSCE do not differ significantly (*p *= .86). Independent samples t-tests on overall exam scores revealed that CPCP students (*M *= 67.83, SD = 6.4) scored higher than the control group (*M *= 63.0, SD = 5.85). At an alpha level of .05, this difference was found to be statistically significant, *t *(48) = 2.08, *p *= .043 (two-tailed).

Analyses of the subsections (history taking, physical exam, medical knowledge, and patient education) show a trend toward better performance in CPCP students than in controls, especially in the patient education section (*p *= 0.097), but these differences were not statistically significant.

## Discussion

The use of blended learning is not new, but in the past it consisted primarily of didactic sessions or small groups to bring students in disparate clinical environments together. Using a computer-based model incorporating adult learning principles allows more student flexibility and can present material that simulates actual clinical encounters. Students can work through the material in the way that best suits their learning styles.

Creating online materials allows for a level of creativity absent from writing syllabi or paper cases. We found that the variety of learning activities we could construct was only limited by our imaginations. Striving for the maximum interactivity allowed us to actively engage the students, and their feedback shows that they found the experience to be educationally valuable.

Based on student feedback surveys, we found that the online curriculum was well accepted, despite the extra time students spent to complete it. This likely relates to the students' ability to work on new skills and directly apply them to their work with patients. Having an interactive virtual chart allowed students to practice note and prescription writing on their own, away from the time pressures of the clinical setting. Likewise, seeing approaches to challenging patient interviews online gave students the opportunity to increase their comfort level with such patients and to reflect on their own skills outside of real clinical encounters.

More advanced students could use the curriculum to explore common primary care complaints and their differential diagnoses, tests, and treatments. Some students, however, commented that the information in Module 3 was presented at too basic a level. Reviewing the content with an eye toward its applicability to a wider range of diagnostic abilities might have improved the experience for clinically advanced students.

Although all Case medical students are issued identical laptop computers on matriculation, there were still technologic issues. Some students with slow internet connections at home had trouble viewing the streaming video and listening to the streaming audio. With the increasing availability and affordability of high-speed internet connections, this problem should become less pressing. For medical schools that don't provide or require specific computer specifications, however, care must be taken in creating content that can be used by all students.

There are strengths and limitations to all educational formats, and blended learning is no exception. We have highlighted the ability of an online curriculum to enhance clinical learning by taking advantage of interactive technology and standardizing student experiences in a way that is not place or time dependent. One obvious weakness is the distant nature of the interactions between students and teachers when communicating via discussion board or email. This is balanced, in CPCP, with the face-to-face interactions that the students have weekly with their preceptors. There is also a limited amount of flexibility in the content delivered to individual students. We addressed this by including as many links to further reading and related information as possible so each student could find content that suited his/her level of expertise.

The major limitation to this study is the difficulty in measuring behavioral outcomes. Due to the small number of control students as well as the large number of other clinical experiences during the two years of CPCP participation that could have influenced student performance on the clinical exam, sweeping conclusions are not possible. We were encouraged to see that PCT students performed well on the exam when compared with their peers and that there was a trend toward superior performance in patient counseling and a significant difference in overall performance. The strength of the blended learning model may not be reflected as clearly in performance data as it is in the feedback the students provide on their ability to use the knowledge gained to improve the actual care of patients.

In addition, when Case underwent a major curricular revision in 2006, CPCP and the online curriculum were expanded to include all second year medical students. This expansion of CPCP from an elective for primary care track students to a mandatory course further exemplifies the success of this program.

## Conclusion

In summary, a blended learning course using an interactive online curriculum to augment clinical learning in a longitudinal primary care preceptorship was successful in engaging second and third year medical students. Participants rated the content and the format highly and found that they could use the information gained in their real clinical lives. We attribute this success to the creative use of computer technology and the practical nature of the material. Medical educators should consider the blended learning format as they strive to standardize the clinical learning of their students.

## Competing interests

The authors declare that they have no competing interests.

## Authors' contributions

LL was the director of the CPCP program and led the development of the web-based modules. She analyzed the student feedback data, contributed significantly to the development of the Generalist OSCE exam and was involved in the analysis of the student scores, and was a major author of this manuscript. MS became the director of the CPCP program after LL left Case Western Reserve University. She contributed to the analysis of student feedback and Generalist OSCE data and was a major author of this manuscript. BB was the educational designer of the CPCP curriculum and created the structure for delivering the content and for collecting student feedback data. She contributed to the writing of this manuscript and in reviewing it carefully for important content. PG was the administrative head of the Primary Care Track program that included CPCP. She contributed significantly to the implementation of the Generalist OSCE exam and to the collection of data on student performance. She contributed to this manuscript by reviewing it carefully for intellectual content.

## Pre-publication history

The pre-publication history for this paper can be accessed here:


